# Radiomics in Glioblastoma: Current Status and Challenges Facing Clinical Implementation

**DOI:** 10.3389/fonc.2019.00374

**Published:** 2019-05-21

**Authors:** Ahmad Chaddad, Michael Jonathan Kucharczyk, Paul Daniel, Siham Sabri, Bertrand J. Jean-Claude, Tamim Niazi, Bassam Abdulkarim

**Affiliations:** ^1^Division of Radiation Oncology, Department of Oncology, McGill University, Montreal, QC, Canada; ^2^Department of Pathology, McGill University, Montreal, QC, Canada; ^3^Research Institute of the McGill University Health Centre, Glen Site, Montreal, QC, Canada; ^4^Department of Medicine, McGill University, Montreal, QC, Canada

**Keywords:** brain, cancer, diagnosis, glioma, MRI, radiomics, glioblastoma

## Abstract

Radiomics analysis has had remarkable progress along with advances in medical imaging, most notability in central nervous system malignancies. Radiomics refers to the extraction of a large number of quantitative features that describe the intensity, texture and geometrical characteristics attributed to the tumor radiographic data. These features have been used to build predictive models for diagnosis, prognosis, and therapeutic response. Such models are being combined with clinical, biological, genetics and proteomic features to enhance reproducibility. Broadly, the four steps necessary for radiomic analysis are: (1) image acquisition, (2) segmentation or labeling, (3) feature extraction, and (4) statistical analysis. Major methodological challenges remain prior to clinical implementation. Essential steps include: adoption of an optimized standard imaging process, establishing a common criterion for performing segmentation, fully automated extraction of radiomic features without redundancy, and robust statistical modeling validated in the prospective setting. This review walks through these steps in detail, as it pertains to high grade gliomas. The impact on precision medicine will be discussed, as well as the challenges facing clinical implementation of radiomic in the current management of glioblastoma.

## Introduction

Glioblastoma (GBM) is the most common astrocytic primary brain malignancy, with an annual incidence of 2–3 cases per 100,000 adults in North America and Europe (1, 2). The standard of care for newly diagnosed GBM combines maximum safe resection followed by chemo-radiation and adjuvant courses of temozolomide (TMZ) (3). The median overall survival is poor at 14.6 months and 5-year survival rates are under 10% following standard of care treatment. If patients tolerate the chemoradiotherapy without progression, they may be considered for tumor-treatment fields. Even in this setting, the survival is still limited at a median of 20.9 months (4). Given these poor outcomes, there is hope that up-and-coming therapies will show benefit in the randomized setting (5, 6). It will be essential to ascertain which patients can benefit from these therapies, highlighting the need for efficacious tools to offer personalized medicine.

Magnetic resonance imaging (MRI) is the preferred imaging modality for both the diagnosis and monitoring of central nervous system (CNS) malignancies (7). It provides a massive amount of information to clinicians. Unfortunately, clinicians are typically restricted to qualitative descriptors or subjective quantitative assessments to articulate changes in imaging. The resulting clinical evaluations have a significant potential for bias.

Clinicians immensely value non-invasive approaches that can direct patients to the correct therapeutic approach in an objective fashion. This begins at diagnosis, where various molecular factors differentiate the diagnosis between low-grade glioma, high-grade glioma, or GBM (8). Such factors may also be predict the efficacy of a systemic agent (9, 10). This information requires tissue, introducing patient morbidity, an additional procedure, and a variety of expensive molecular assessments.

Radiomics has demonstrated remarkable progress in demonstrating that it may be a tool that can derive this information. Radiomics is a field of biomedical imaging using advanced non-invasive assessments of complex imaging characteristics within the MRI images that are too complex for a human to appreciate (11–14). These characteristics are known as *features*. Imaging features have been associated with a CNS tumor's histological features (14), progression (15) grade (16), or even overall survival (17–21). Radiomics analysis thus hosts a major role in producing novel non-invasive biomarkers acquired from a test—MRI—that is already routinely acquired from patients as part of the standard of care.

## Radiomics Methodology

**Figure 1:**
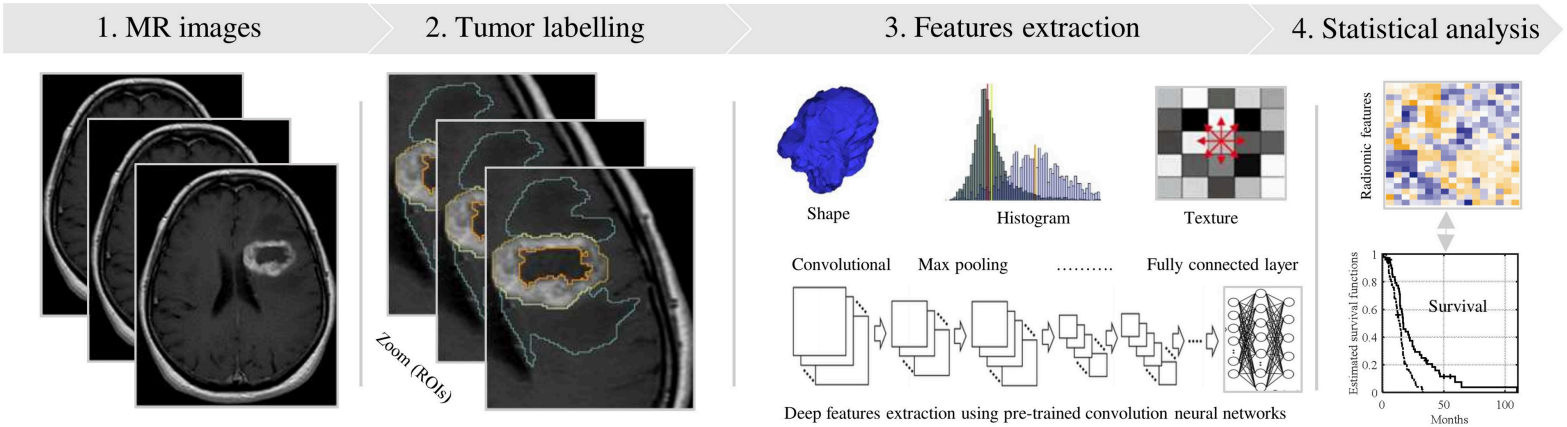
Standard pipeline of the radiomics analysis. (1) MR Image acquisition with a standardization. (2) Tumor labeling viewing in 3D (e.g., red, yellow and cyan contours). (3) Radiomic features extraction using shape, texture and convolution neural network techniques. (4) Statistical analyses, based significance test and classifier models, to identify relevant features for predicting the clinical outcome.

A standard pipeline of radiomic analysis has been described by several studies in the past (Figure 1) as mentioned previously by several studies (12, 13, 19, 21–23). This review discusses recent studies in the development of MRI-based radiomics analysis in relationship to this pipeline. For CNS malignancies, the literature discusses the most significant cause of diagnostic and management dilemmas—low and high-grade glioma. To facilitate an understanding of the process, there are sections on the: (1) preprocessing and image acquisition for developing a radiomic model; (2) segmentation/labeling of the cancer; (3) identification of relevant features types that may relate to the molecular properties of the tumor (14, 24) and (4) statistical modeling to describe a radiomic profile's relationship with a clinical outcomes. Given the number of variables at each step, collaboration is essential. Radiologists and oncologists must ensure that the appropriate regions are being assessed and the right questions are being asked. Molecular scientists must communicate the relevant genetic and proteomic characteristics that will influence a patient's clinical course. Engineering teams must determine what information can be reliably extracted from the images and then adapt the machine learning to fashion a reliable model. Consultation with statisticians will allow for a methodological approach allows for a potentially statistically significant solution.

### Image Acquisition

MRI radiomics has repeatedly shown the ability to differentiate low and high-grade glioma, which have different management strategies (https://www.nccn.org/) and a remarkably different prognosis (25–28). One reason this data can be more rapidly generated is that there is a wealth of clinical information available—glioma patients have regular MRIs throughout their lives. However, reproducibility is a significant issue at different stages of the radiomics pipeline. The issues begin at image acquisition. Different academic groups acquire their MRI images to different settings at the first step of the pipeline. This is one reason that radiomic analysis collaboration has been limited between research groups. Standardization offers a rational solution to overcome this barrier.

### Standardization

Potential variations in images are often secondary to the MRI scanner model, including image resolution (i.e., pixel size and slice spacing), image contrast, slice thickness, patient position, and further variations introduced by different reconstruction algorithms. When generating or applying a radiomics model, standardization must occur so the data can be assumed that it was extracted from similar settings. To accomplish this, volume datasets are usually re-sampled to a common voxel resolution of 1 mm^3^ and an image size of 256^3^ (or 512^3^) voxels.

A common further step is normalizing the intensities within each volume image to the [0,1] or [0,255] range. Less commonly adopted normalization approaches have included gaussian and Z-score normalization. For example in Ellingson et al. (29), Gaussian normalization was the best normalization technique for image intensity correction. The need for standardization would be reduced if radiomic analysis could be performed with data acquired at a single geographical site. However, a single site would only provide a limited dataset. Thus, several studies have augmented their datasets through the use of multiple sites and an imputation technique to facilitate standardization (30).

The lack of standardization is a recognized problem. The *Quantitative Imaging Biomarker Alliance* offers an expert consensus after reviewing the available data. This group offers insightful guidelines for standardization that should be heavily considered in present and future studies. Such guidelines will be dynamic. Radiomic features may change from site to site or have new ways to be extracted or MRI image acquisition may change. Standardization in either of these contexts will a challenge in the future. Ongoing communication between institutions and robust reporting of new methodological approaches will be essential to groups studying radiomics.

### Segmentation of Brain Tumors

Accurate labeling of brain tumors in the images is required for radiomic analysis. It first involves defining the tumor volume, known as the *region of interest* (ROI), so it can then have its radiomic features extracted. The act of employing clinical, pathological and imaging features to mark out the ROI on the two-dimensional MRI images is called either the *segmentation* or *labeling* process. Segmentation is performed by clinicians—typically a radiologist or oncologist. The process is subject to inter-rater variability, as the ROI definition will inevitably differ between clinicians. An approach to overcome this variation is different clinicians each generating their own ROI. The geographical regions common between the different ROIs is considered the true tumor mask. This tumor mask is then matched with the corresponding brain images to then extract the imaging features (i.e., radiomic features).

Since MRI generates several image sequences, the registration step involves matching the mask to the relevant MR series (ex. T1 weighted, T1-post contrast, T2 weighted, FLAIR), a well-described process (20, 31). Many tools used to delineate the ROI, such as the publicly accessible 3D Slicer (32), require slice by slice labeling on each series to ensure accuracy and precision (21). For efficiency and to minimize both inter- and intra-user variability, several studies have explored segmentation to all relevant MRI sequences without registration across the sequences (33). Registration distortions between MRI series may limit this approach (34). Distortion could cause incorrect localization of the ROI, directing the radiomic analyses to the incorrect MRI-defined anatomy. More investigation is required to allow for the trans-sequence application of user-generated segmentation data.

To overcome user variability in registration, (semi-)automated segmentation has been explored in various studies (35–38). Strong signals for a successful model, a promising Dice Similarity Coefficient (DSC) of 80%, have been reported with fully automated segmentation based on an adaptive algorithm with multi-level of thresholding (38). When deep learning radiomics (DLR) was applied to multiple tumor regions, the ability to label the tumor subregions achieved a DSC of 90% (35). DLR has become a success story for machine learning integral to limiting user variability. The use of DLR's convolutional neural networks (CNNs) to the various steps of the radiomics pipeline is elegantly described elsewhere (39). As to fully automated segmentation, further validation is required. Success here could enable the rapid integration of radiomics into personalized medicine.

### Radiomic Features Extraction

Extracting radiomic features is the first step in analyzing the segmented image. The features themselves are measures of the heterogeneity within the ROI (40). The degree to which these different features are present is a radiomics feature cluster, perhaps better conceptualized as an ROI's radiomic signature. There are different types of features, the most common and presently relevant are outlined in the Table 1.

**Table 1:** Features extraction techniques used in radiomic analysis.

**Histogram features:** These are first-order statistics computed from image's histogram of voxel/pixel intensities. Histogram features (e.g., average, standard deviation, skewness, kurtosis, energy and entropy) encode the voxel intensities and the shape of the data's distribution (41, 42). In non-CNS malignancies, these features have been associated with histological features, subtype and grade (43, 44).
**Texture features:** Texture features use second order statistics to characterize the spatial relationship between voxel intensities, describing the local spatial arrangement of intensities in the image. The features encode several matrices that represent the special intensity distribution in several ways. Not included in the list below are also texture features based on several conventional techniques that have been predictive of clinical outcomes, such as: as scale-invariant feature transform (SIFT), histogram of oriented gradients (HOG), fractal texture analysis (FTA) and local binary patterns (LBP) (45–47). Elsewise, the most common texture features are: *Gray-level co-occurrence matrix* (GLCM)—the most commonly used texture feature. Considering only voxels within a specific range of gray values, it produces a matrix of the spatial relationships of pairs of voxels (48). *Joint intensity matrix* (JIM)—evaluates the spatial relationships of pairs of voxels within given intensity ranges across different MRI different sequences. This is in contrast to GLCM, which is restricted to a single MRI sequence (21). *Neighborhood gray-tone difference matrix* (NGTDM)—a description of the differences in signal intensity, or gray-tone, between each voxel and its neighboring voxels (49). It has been used in several topics of images analysis and classifications (45). *Neighboring gray-level dependence matrix* (NGLDM)—Similar to NGTDM, is computed from the gray tone relationship between every element in the image and all of its neighbors at a certain distance (50, 51). *Gray-level run length matrix* (GLRLM)—A matrix of all the voxels within the same gray level value (52).
**Multiscale texture features:** These features have been derived from filters, such as the Laplacian or Gaussian filter (53), that serve as a generic differential operator. Multiscale texture features provide an excellent description of local image variations, such as edges or blobs. The ROI's image is filtered in a multiscale way—from fine to coarse texture—that can be quantified by parameters like entropy (31, 54). The wavelet decomposition of an image generates multiscale texture images based on multiband frequencies, a radiographic characteristic called a *detail*. Each of these bands has a scale of the texture inside the image. A quantifier function then evaluates the texture of the images, using the resultant value as an input for a classifier model (42, 55).
**Deep features:** These features are derived from deep neural networks, the process of which is well-described in a recent review (56). To accomplish this, a pre-trained network must be established prior to texture extraction. As a case study from the literature, (1) ImageNET was pretrained to identify textures, (2) the CNN analyzed a fully connected layer of ImageNet, deriving 4,096 texture features, then (3) these features were used an input for a classifier model, which could also incorporate a CNN (as described in this review's Radiomics Analysis step) (39). However, CNNs require numerous examples to develop a reliable model. In general, studies implementing CNNs require more patients than the number of features being analyzed. Achieving this sample size can be a challenge, so alternative methods of model generation are needed for many studies. One such example reported the conditional entropy from a texture of the CNN's feature map. This was a reliable alternative when implemented into a random forest classifier, instead of another different standard CNN model (57).
**Shape features:** Shape features describe the 3D (or 2D) geometrical composition of the ROI considered the size (e.g., volume), form (e.g., sphericity, solidity, major length axis) and tumor location. As with traditional radiological assessment, shape is a characteristic that does relate to tumor characteristics with radiomics as well (19, 58, 59).

### Feature-Analyses

Once the features have been extracted, statistical modeling can highlight relationships between the extent a given feature is present and a clinical characteristic. There are various methodologies to analyze this, including minimizing the number of features likely to contribute to the statistical analysis. Feature selection methods (60) or reducing dimensionality in another fashion can accomplish this minimization. This has included sorting features by their minimum redundancy maximum relevance, mutual information, principal component analysis feature rank or the importance of features in other classifier models (31, 61–65). Once the features that are potentially relevant for analysis are determined, they are typically subject to assessments of their significance (e.g., Wilcoxon test, Kruskal-Wallis, log-rank, etc.) and correlation (e.g., Spearman rank, Pearson). These forms of *univariate analysis* determine if a feature is a significant predictor for the selected clinical outcome, with significance typically being defined as either a *p* < 0.01 or 0.05. The *p*-values should be corrected by the Bonferroni or Holm-Bonferroni procedure to limit the influence of random chance, including the false discovery rate (21, 60, 66).

### Multivariate Analysis and Model Building

Multivariate analysis fills an essential role in separating seemingly relevant features on univariate analysis from those that are likely *independent* predictors for the clinical outcome being assessed for. This is a critical step, limiting non-contributory features from influencing our eventual final statistical model (67). Once these features are selected from multi-variate analysis, the radiomics team must determine how many of their finite number of clinical cases will be used to produce/train their model and how many need to be reserved to validate the model.

Increasing the size of the training cohort will increase the model's accuracy. Thus, typically 70–80% of the dataset is used for the training stage. Alternatively, if an external dataset is available, then all the datasets can train the model. This is the preferred scenario, allowing for a demonstration of external validity. If the datasets are limited in size, k-folds cross-validation can mitigate some of the statistical concerns (31, 68, 69).

Machine learning changes the available options. If unsupervised, the program can utilize different methods (e.g., k-means, nearest neighbors) to partition the features into different groups, then compare the relationships of the different features within their group—*not* the clinical data. After this is completed, the ability of the different groups to predict the clinical outcome is assessed, even though the clinical data did not contribution to the model's development (70). In comparison, supervised machine learning techniques (e.g., support vector machine, Bayes model, neural network nearest neighbors, random forests) will place varying numbers of the pre-determined relevant features into groups. Then their relative contribution to the model's ability to predict for the clinical outcome is altered until the most reliable combination of weightings is determined. Random forest classifier is a simple model that automatically selects the relevant features. Furthermore, random forest has shown the great ability to predict for survival (71) and endure an imputation technique to account for censored patients (31). Alternatively, the least absolute shrinkage and selection operator (LASSO) Cox regression model has also been reported reliably predict for survival in glioma (72, 73).

A third option is semi-supervised machine learning, wherein *some* complete clinical data is provided to the program generating the model, but other data is complete. For example, the program would have a range of radiomic features that it knows correspond to high grade glioma and a range of radiomic features that belong to an unknown clinical entity. Thus, all the dataset is used for a training step. The validation step is then a question if the program can correctly identify the unlabeled data. This process has been used to suggest brain tumor histology and prognosis (74).

## Progress of Radiomics in GBM

Radiomics has provided key insight into critical features of GBM, as advanced radiomic analysis seek to establish reliable associations between key clinical features and those features derived from images. For example, radiomics has been used to predict for clinical, proteomic (e.g., Ki-67 expression), genomic (e.g., IDH1 status) and transcriptomic characteristics (75–77). This evolution of the radiomics field has been titled multi-omics or radiogenomics, dependent on the source (21, 78–80). This will be part of the future of radiomics, as these details are pertinent to physicians due to their influence on treatment and prognosis (8). In addition, recent advancements have been made in defining radiomic subtypes. By utilizing T1 and FLAIR sequencing, researchers were able to define three distinct imaging subtypes—rim enhancing, irregular and solid. Each subtype represents a distinct phenotype enriched in unique molecular alterations such as MGMT methylation and EGFRvIII mutations (37). Continued advancements in defining tumor heterogeneity using imaging features may offer a complimentary means with which to characterize GBM and provide personalized treatments for patients.

Radiomics analysis has the capacity to answer critical questions facing clinicians such as the discrimination between pseudoprogression and progressive disease in GBM patients. For example, combining the *diffusion tensor imaging* and *dynamic susceptibility contrast* MRI features can improve accuracy treatment response and may aid in individualized treatment of patients with GBM (81). Recently, a deep radiomics model used the MR images with clinical features demonstrate the capacity to predict the PsP from progression for patients with GBM (82). While, another study showed that the radiomics analysis is not able to distinguish between true-progression and PsP (83). However, many of these steps exist in an early developmental stage. Combining all such information into an artificial intelligence model would be a promising direction to advance personalized medicine.

## Intratumoural Heterogeneity and Radiogenomics

Perhaps the greatest utility of radiomics in the management of gliomas lies in the application of radiogenomics. Radiogenomics implements radiomics analysis to predict specific genetic characteristics. Classically, gliomas have been managed based on their grade—a histopathological characterization made by specialized physicians (neuropathologists) to articulate the likely behavior of the malignancy. Over the past two decades, molecular assessment of the tumor's genome, protein expression, and epigenetic state have become more common as the relevance of these features to outcome and/or therapeutic response is being increasingly understood (84). Given the relative abundance of high quality MRI data which accumulates over time during standard of care for glioma patients (85–87) radiomics offers a potentially efficient and non-invasive method of tumoral evaluation (37, 88, 89). Indeed, recent efforts have generated radiomic signatures to predict the majority of information sought by classical histopathological and modern molecular assessments including: isocitrate dehydrogenase mutations (79, 90–92), 1p/19q codeletion loss of heterozygosity (24, 92, 93), O^6^-methylguanine-DNA methyltransferase promoter methylation (45, 94) and ATRX mutations (95). This has culminated in recent findings demonstrating a conserved radiomic signature can predict CD8+ T-cell infiltration and response to immunotherapy (96).

However, intratumoral heterogeneity significantly confounds both molecular and histopathological assessments as the entirety of a tumor cannot be assessed by neuropathologists. Disparate clonal populations may be minimally represented in histopathological sampling introducing sampling errors and limiting relevance for informing treatments (97–101). Radiomics offers an opportunity to overcome this limitation as analysis is performed upon the complete tumor enabling spatial mapping of distinct genetic features. In addition, radiomics offers the means to provide quantitative values (e.g., % of tumor mutated) rather than binary designations (e.g., mutant or not) to describe molecular features which may have important implications for predicting response to therapies. Utilizing co-clinical models, researchers are starting to establish radiomic signatures which are closely associated with specific molecular features in an attempt to describe intratumoral heterogeneity (102). Further development of pre-clinical models and correlation with clinical datasets will be essential to drive this field forward toward improving the utility of radiomics for diagnosis in GBM.

## Future Radiomics

Radiomics needs massive amounts of biomedical data, so-called “Big data (103),” to validate it's deep-learning approaches and expanding applications. The development of strong public datasets has empowered these approaches, with such initiatives including The Cancer Genome Atlas (TCGA) (85), The Cancer Imaging Archive (86), and The Quantitative Imaging Network (87). However, there is still the barrier of segmentation—such as acquiring clinician input to identify the relevant ROIs. While the clinician will still be sought as the gold standard, deep-learning strategies have the potential to define ROIs without the bias of human segmentation (104). To accomplish this, even larger datasets will be required—further emphasizing the need for reliable Big Data. These strategies have begun in part, but developing validated models to all the clinically relevant questions will simply require more data (105, 106).

The potential applications for radiomics is expanding, with logistical and technical challenges needing to be overcome prior to true clinical deployment. We view these as: (1) expanding what is included in and the access to Big Data, (2) establishing common criteria from image acquisition to feature definitions, (3) agreement on the clinical questions that radiomics must address, and (4) developing a clinically implementable and prospectively validated statistical model to answer those questions.

## Conclusions

This review explained how the vast amount of radiological data not used by the clinicians managing CNS malignancies can be used to generate radiological signatures that can predict the characteristics of these brain tumours. In a step-by-step process we outlined how this data can be used to predict for numerous pertinent biological outcomes. With constant progress in deep-learning processes and expanding public access to Big Data, radiomics has the potential to non-invasively address numerous clinical questions or support clinical decision making. There are numerous future directions for radiomics, but a continued focus on ensuring there is public access to large databases of clinical and radiological correlated data will be instrumental to seeing those directions leading to a desirable destination.

## Author Contributions

AC conception and design. AC, PD, and MK drafting the manuscript and review of the literature. MK, PD, BJ-C, TN, SS, and BA critical revision of the manuscript.

### Conflict of Interest Statement

Author PD's fellowship was partly funded by a grant from Varian Medical System. The funder had no role in study design, data collection and analysis, or preparation of the manuscript. The remaining authors declare that the research was conducted in the absence of any commercial or financial relationships that could be construed as a potential conflict of interest.
